# Analysis of Differential Metabolites in Citrus Leaves Infected with Huanglongbing and Screening of Biomarkers for Early Detection

**DOI:** 10.3390/molecules31111873

**Published:** 2026-05-29

**Authors:** Lu Chen, Jiayu Meng, Zilan Shi, Xinhua He, Yang Zhao, Guiying Zhang

**Affiliations:** 1 State Key Laboratory of Conservation and Utilization of Subtropical Agro-Bioresources, Guangxi University, Nanning 530004, China; 16636021984@163.com (L.C.); 18232442756@163.com (J.M.); 18360701356@163.com (Z.S.); honest66222@163.com (X.H.); 2 Guangxi Key Laboratory of Sugarcane Biology, Guangxi University, Nanning 530004, China

**Keywords:** Huanglongbing, Orah mandarin, metabolomics, dual-platform (GC-MS/LC-MS), biomarkers, metabolic pathways

## Abstract

Huanglongbing (HLB), a destructive citrus disease caused by *Candidatus Liberibacter asiaticus* (CLas), cannot be efficiently diagnosed at the early stage via conventional detection methods. Most current metabolomic studies on citrus HLB rely solely on the LC-MS platform and mainly focus on sweet orange, while integrated dual-platform metabolomic research targeting Orah mandarin, the dominant cultivated citrus variety, remains limited. In the present study, GC-MS and LC-MS were combined for the first time, alongside multivariate statistical analysis and pathway enrichment analysis, to systematically characterize metabolomic differences between healthy and HLB-infected Orah mandarin leaves. Suspected citrus leaf samples were collected from two major citrus-producing regions in Guangxi and further confirmed by qPCR detection. A large number of differentially accumulated metabolites were identified, which were primarily enriched in carbohydrate metabolism and amino acid metabolism pathways. Several candidate biomarkers applicable for early HLB diagnosis were also screened out. This study provides reliable scientific evidence and a theoretical foundation for elucidating the pathogenic mechanism of HLB and establishing field-based early diagnosis techniques.

## 1. Introduction

Huanglongbing (HLB), a devastating citrus disease, is caused by the phloem-colonizing bacterium *Candidatus Liberibacter asiaticus* (CLas) [1]. No curative therapies have been available for HLB to date, and disease control relies primarily on preventive measures such as removal of infected trees and chemical suppression of pathogenic inoculum [2]. Conventional HLB detection methods—including field symptom observation [3,4], Polymerase Chain Reaction (PCR) [5], loop-mediated isothermal amplification (LAMP) [6], and modulated chlorophyll fluorescence assay [7]—are limited by low sensitivity for early diagnosis, cumbersome operation, or high cost, thus failing to block disease transmission from asymptomatic citrus trees [8,9].

As a powerful systems biology tool, metabolomics is widely used in plant–pathogen interaction research to identify differentially accumulated metabolites (DAMs) and potential biomarkers. In numerous studies on plant diseases, metabolites have been validated as reliable sources of biomarkers: in the interaction between cashew trees and *Colletotrichum* spp., 12 potential biomarkers were identified via ultra-performance liquid chromatography-quadrupole time-of-flight mass spectrometry in MSE mode (UPLC-QTOF-MSE), among which triene-(17:3)-anacardic acid served as the core biomarker for disease resistance, while catechin and proanthocyanidin isomers acted as susceptibility-related biomarkers [10]; in the tripartite interaction assay of tomato with the phytopathogen Alternaria solani and the beneficial bacterium Bacillus subtilis, metabolites such as quercetin and salvianin were explicitly identified as biomarkers to distinguish different treatment groups [11]. Additionally, pterostilbene, a key metabolite in the phenylpropanoid metabolic pathway, exerts significant inhibitory effects on mycelial growth and spore germination of various Colletotrichum species, thus being recognized as a crucial metabolic biomarker associated with anthracnose resistance [12]. Collectively, these studies demonstrate that metabolic biomarkers can effectively characterize plant resistance/susceptibility status and pathological progression. However, metabolomic studies on HLB remain insufficient [13,14]. Existing research has mainly focused on sweet orange (*Citrus sinensis*) and other citrus cultivars [15], mostly based on a single LC-MS platform [16]. Integrated GC-MS/LC-MS dual-platform metabolomic analysis of citrus samples from multiple regions—especially *Citrus reticulata* × *Citrus sinensis* ‘Orah’ (Orah mandarin), a globally cultivated and economically significant cultivar—is currently lacking, which cannot meet the global demand for early HLB diagnosis.

Guangxi is the largest citrus-producing region in China, where Orah mandarin accounts for over 30% of the total citrus planting area (Department of Agriculture and Rural Affairs of Guangxi, 2022). The annual incidence of HLB in this cultivar reaches 12% (2025), leading to direct economic losses exceeding 1 billion Chinese Yuan (Standing Committee of Guangxi People’s Congress, 2019) [17]. Notably, citrus production areas in Guangxi are mainly concentrated in Nanning and Liuzhou. A field survey was conducted in Nanning, Liuzhou, with 700 suspected HLB leaf samples collected from eight citrus cultivars. GC-MS and LC-MS were used to characterize metabolite profiles in healthy and HLB-infected Orah mandarin leaves. Combined with multivariate statistical analyses (PCA, OPLS-DA), pathogenesis-related DAMs were screened to identify potential biomarkers for early HLB detection. This study provides a scientific basis for field HLB diagnosis and control, and promotes the sustainable development of the citrus industry.

We hypothesized that HLB infection induces distinct and detectable metabolomic alterations in Orah mandarin leaves and that integrated GC-MS/LC-MS metabolomics can identify robust biomarkers for early HLB diagnosis.

The objectives of this study were to: (i) characterize metabolomic differences between healthy and HLB-infected Orah mandarin leaves using integrated GC-MS and LC-MS platforms; (ii) identify differential metabolites and enriched metabolic pathways associated with HLB infection; and (iii) screen potential biomarkers for early HLB detection.

## 2. Results

### 2.1. qPCR Detection of Citrus HLB and Screening of Positive Samples

qPCR detection was conducted on 700 suspected HLB leaf samples from Nanning, Liuzhou, and Guangxi University’s experimental base, identifying 62 HLB-positive plants (Table 1). No positive plants were detected in Seedless Orah mandarin and Golden Autumn mandarin. Positive sample distribution: 11 from Satsuma mandarin (sample code WZ), 8 from Sweet orange (sample code TC), 11 from Shatangju mandarin (sample code ST), 12 from Nanfeng tangerine (sample code NF), 9 from Orah mandarin (sample code WG), and 11 from Tangelo (sample code JY). Survey results showed mild HLB incidence in Wuming orchards, with more severe spread in Liuzhou. As Orah mandarin is the staple cultivar of Guangxi’s citrus industry, studying its HLB metabolic mechanisms and biomarkers has greater translational value. Accordingly, all subsequent widely targeted metabolomic analyses in this study were selectively performed using Orah mandarin samples, and the detailed sample grouping information is provided in Supplementary Table S1.

### 2.2. LC-MS-Based Metabolomic Profiling and Differential Metabolite Identification

**Figure 1:**
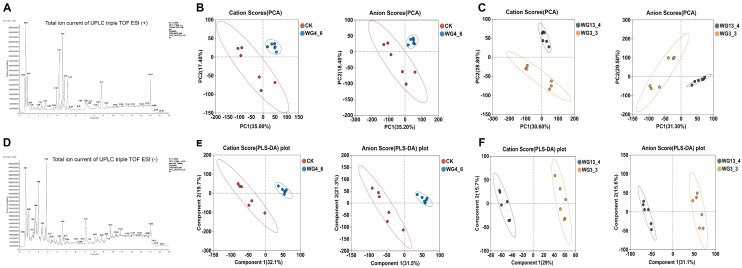
Metabolic profiling of Orah leaf samples via UPLC-triple TOF mass spectrometry. (**A**) Total ion current (TIC) chromatogram of UPLC-triple TOF MS in positive electrospray ionization (ESI+) mode, displaying well-resolved peaks and uniform signal distribution. (**B**) Principal component analysis (PCA) score plots for cation (left panel) and anion (right panel) metabolites in diseased (WGL_6) vs. healthy (CK) Orah leaves. Samples from the two groups were completely segregated within the 95% confidence interval (represented by ellipses), indicating distinct intergroup metabolic differences. (**C**) PCA score plots for cation (left panel) and anion (right panel) metabolites between infected Orah leaf samples from distinct orchards (WG13_4 vs. WG3_3). (**D**) TIC chromatogram in negative electrospray ionization (ESI−) mode, with similarly high peak resolution and consistent signal distribution. (**E**) Orthogonal partial least squares-discriminant analysis (OPLS-DA) score plots for cation (left panel) and anion (right panel) metabolites in diseased vs. healthy leaves. (**F**) OPLS-DA score plots for cation (left panel) and anion (right panel) metabolites between infected samples from different orchards.

LC-MS annotated 1229 total metabolites. TICs in positive and negative ion modes showed well-resolved peaks and uniform distribution (Figure 1A,D).

PCA revealed cumulative explanation rates (R^2^X) of 0.524 (positive) and 0.536 (negative ion mode) for diseased vs. healthy Orah leaves (Figure 1B), and 0.594 (positive) and 0.608 (negative ion mode) for infected samples from distinct orchards (Figure 1C). Both groups were completely segregated within the 95% confidence interval, indicating significant intergroup metabolic differences.

OPLS-DA further enhanced intergroup discrimination. For diseased vs. healthy leaves, OPLS-DA model parameters were R^2^X = 0.518, R^2^Y = 0.985, Q^2^ = 0.845 (positive) and R^2^X = 0.527, R^2^Y = 0.938, Q^2^ = 0.83 (negative ion mode), indicating robust predictability (Figure 1E). For infected samples from different orchards, OPLS-DA parameters were R^2^X = 0.447, R^2^Y = 0.994, Q^2^ = 0.867 (positive) and R^2^X = 0.467, R^2^Y = 0.994, Q^2^ = 0.892 (negative ion mode) (Figure 1F).

To avoid model overfitting and verify statistical reliability, all established OPLS-DA models were subjected to 200-times permutation tests and CV-ANOVA validation. The results showed that the intercept of Q^2^ was negative, and significant differences were observed in the CV-ANOVA analysis, which confirmed that all constructed models were valid and free from overfitting.

With VIP > 1 and *p <* 0.05, 67 differential metabolites were identified between diseased and healthy leaves (31 positive (Table S2), 36 negative ion metabolites (Table S3)), and 76 differential metabolites were detected in infected samples from different orchards (38 positive (Table S4), 38 negative ion metabolites (Table S5)).

### 2.3. LC-MS-Based Screening of Potential Biomarkers

**Figure 2:**
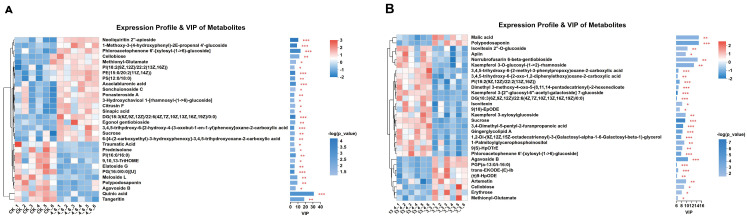
Expression profiles, VIP scores, and statistical significance of differentially abundant metabolites (DAMs) in Orah leaf samples. (**A**) Integrated visualization of the top 30 DAMs between Huanglongbing (HLB)-infected and healthy Orah leaves: hierarchical cluster heatmap (left panel), variable importance in projection (VIP) scores (middle panel), and −log_10_ (*p*-value) (right panel). (**B**) Integrated visualization of DAMs in HLB-infected Orah leaves from distinct orchards: hierarchical cluster heatmap (left panel), VIP scores (middle panel), and −log_10_ (*p*-value) (right panel). Cluster analysis reveals substantial inter-orchard differences in the abundance of these DAMs. Bar color intensity reflects the significance of differential metabolite accumulation, corresponding to the *p*-value: a smaller *p*-value yields a larger −log_10_(*p*-value) and a darker color. Asterisks indicate levels of statistical significance: * *p* < 0.05, ** *p*< 0.01, *** *p* < 0.001.

The top 30 differential metabolites between diseased and healthy Orah leaves are shown in Figure 2A. Cluster analysis indicated that metabolites of HLB-infected samples were mainly concentrated in high-expression regions, while those of healthy samples were in low-expression regions, suggesting higher contents of differential metabolites in infected leaves. Four potential biomarkers were screened: neoglycyrrhizin 2′-apigenin glucoside, 1-methoxy-3-(4-hydroxyphenyl)-2E-propenyl 4′-glucoside, acetophenone 6′-[xylosyl-(1→6)-glucoside], and quinic acid.

For diseased Orah leaves from different orchards, cluster analysis revealed significant differences in differential metabolite contents between orchards (Figure 2B). Two potential biomarkers were identified: kaempferol 3-O-glucosyl-(1→2)-rhamnoside and sucrose.

### 2.4. LC-MS-Based KEGG Pathway Analysis

**Figure 3:**
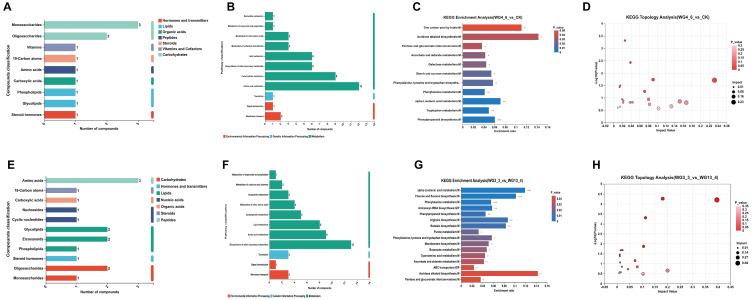
KEGG Brite classification and metabolic pathway enrichment of differentially abundant metabolites (DAMs) in Orah leaf samples. (**A**) KEGG Brite (Level 1/2) compound classification of DAMs between Huanglongbing (HLB)-infected (WG4_6) and healthy (CK) Orah leaves. The bar length represents the number of compounds per category. (**B**) KEGG Brite (Level 1/2) metabolic pathway classification of DAMs for WG4_6 vs. CK. (**C**) KEGG enrichment analysis of DAMs for WG4_6 vs. CK (adjusted *p <* 0.05). The color gradient and bar length represent *p*-value and enrichment ratio, respectively. (**D**) KEGG topology analysis of DAMs for WG4_6 vs. CK, integrating *p*-value (color gradient) and pathway impact value (size of dots) to evaluate the biological importance of enriched pathways. (**E**) KEGG Brite (Level 1/2) compound classification of DAMs between HLB-infected Orah leaves from distinct orchards (WG3_3 vs. WG13_4). (**F**) KEGG Brite (Level 1/2) metabolic pathway classification of DAMs for WG3_3 vs. WG13_4. (**G**) KEGG enrichment analysis of DAMs for WG3_3 vs. WG13_4 (adjusted *p <* 0.05). (**H**) KEGG topology analysis of DAMs for WG3_3 vs. WG13_4, visualizing pathway importance via *p*-value (color gradient) and impact value (dot size). Asterisks denote levels of statistical significance: * *P*/FDR < 0.05, ** *P*/FDR < 0.01, *** *P*/FDR < 0.001.

KEGG Brite Level 1 categories for diseased vs. healthy leaves included carbohydrates, hormones and transmitters, lipids, organic acids, peptides, steroids, vitamins, and cofactors. Level 2 categories were dominated by monosaccharides and oligosaccharides (Figure 3A). KEGG metabolic pathways (Level 1) included metabolism, environmental information processing, and genetic information processing, with amino acid metabolism (12 metabolites) and carbohydrate metabolism (9 metabolites) as the top Level 2 categories (Figure 3B, Table S6). After MapMan classification (*p <* 0.05), differential metabolites were assigned to 11 significantly enriched pathways, with phenylalanine metabolism, phenylpropanoic acid biosynthesis, α-linolenic acid metabolism, and tryptophan metabolism showing the most significant enrichment (Figure 3C,D, Table S7). For diseased leaves from different orchards, KEGG Brite Level 1 categories included 7 classes, with amino acids as the most annotated metabolites (Figure 3E). KEGG metabolic pathways (Level 1) covered environmental information processing, genetic information processing, and metabolism, with amino acid metabolism and biosynthesis of other secondary metabolites as the top Level 2 categories (Figure 3F, Table S8). Differential metabolites were involved in 16 pathways; phenylalanine metabolism, aminoacyl-tRNA biosynthesis, and phenylpropanoic acid biosynthesis were most significantly enriched (*p* < 0.05) (Figure 3G,H, Table S9).

### 2.5. GC-MS-Based Metabolomic Characteristics and Differential Metabolite Identification

**Figure 4:**
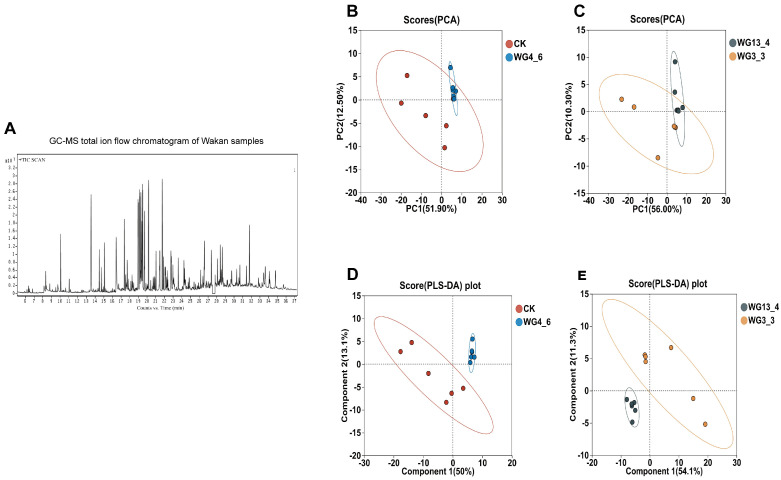
GC-MS-based metabolic profiling of Orah leaf samples. (**A**) Total ion current (TIC) chromatogram of Orah leaf samples acquired via GC-MS, displaying well-resolved peaks. (**B**) Principal component analysis (PCA) score plot for Huanglongbing (HLB)-infected (WG4_6) vs. healthy (CK) Orah leaf samples. (**C**) PCA score plot for HLB-infected Orah leaf samples from distinct orchards (WG3_3 vs. WG13_4). (**D**) Orthogonal partial least squares-discriminant analysis (OPLS-DA) score plot for WG4_6 vs. CK samples. (**E**) OPLS-DA score plot for WG3_3 vs. WG13_4 samples.

GC-MS annotated 175 total metabolites, with well-resolved peaks in TIC (Figure 4A). PCA showed a cumulative explained variance (R^2^X) of 0.644 for diseased vs. healthy Orah leaves (Figure 4B), and R^2^X = 0.663 for infected samples from different orchards (Figure 4C), with distinct intergroup separation.

OPLS-DA model parameters for diseased vs. healthy leaves were R^2^X = 0.631, R^2^Y = 0.914, Q^2^ = 0.686 (good predictability) (Figure 4D). For infected samples from different orchards, model parameters were R^2^X = 0.653, R^2^Y = 0.915, Q^2^ = 0.729 (good predictability), with distinct separation between orchards (Figure 4E).

All OPLS-DA models were validated by 200 permutation tests and CV-ANOVA analysis. The verification results confirmed that these models had no overfitting tendency and possessed satisfactory robustness and reliable predictive capability.

With VIP > 1 and *p <* 0.05, 49 differential metabolites were identified between diseased and healthy Orah leaves (Table S10), and 41 differential metabolites were detected in infected samples from different orchards (Table S11), involving organic acids, carbohydrates, amino acids, and alcohols.

### 2.6. GC-MS-Based Screening of Potential Biomarkers

**Figure 5:**
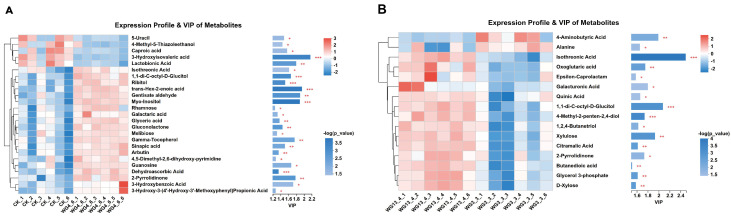
Expression profiles, VIP scores, and statistical significance of differentially abundant metabolites (DAMs) in Orah leaf samples. (**A**) Integrated visualization of the top 30 DAMs between Huanglongbing (HLB)-infected (WG4_6) and healthy (CK) Orah leaves, comprising a hierarchical cluster heatmap (left panel), variable importance in projection (VIP) scores (middle panel), and -log_10_(*p*-value) (right panel). (**B**) Integrated visualization of DAMs in HLB-infected Orah leaves from distinct orchards (WG13_4, Liuzhou Orchard vs. WG3_3), including a hierarchical cluster heatmap (left panel), VIP scores (middle panel), and −log_10_(*p*-value) (right panel). Cluster analysis reveals higher metabolite abundances in samples from Liuzhou Orchard (WG13_4). Bar color intensity reflects the significance of differential metabolite accumulation, corresponding to the *p*-value: a smaller *p*-value yields a larger −log_10_(*p*-value) and a darker color. Asterisks indicate levels of statistical significance: * *p* < 0.05, ** *p*< 0.01, *** *p* < 0.001.

The top 30 differential metabolites between diseased and healthy Orah leaves are shown in Figure 5A. Cluster analysis indicated higher contents of differential metabolites in diseased leaves. Among these, 5-uracil, 4-methyl-5-thiazoleethanol, hexanoic acid, 3-hydroxyisovaleric acid, and lactic acid were significantly decreased in infected samples, while 1,1-di-C-octyl-D-glucitol, ribitol, trans-hex-2-enoic acid, gentisaldehyde, and inositol were substantially accumulated. Four potential biomarkers were screened: 3-hydroxyisovaleric acid, 1,1-di-C-octyl-D-glucitol, trans-hex-2-enoic acid, and inositol.

For diseased Orah leaves from different orchards, cluster analysis revealed higher metabolite contents in Liuzhou Orchard samples (Figure 5B). Three potential biomarkers were identified: isothreonic acid, 4-methyl-2-pentene-2,4-diol, and 1,1-di-C-octyl-D-glucitol.

### 2.7. GC-MS-Based KEGG Pathway Analysis

**Figure 6:**
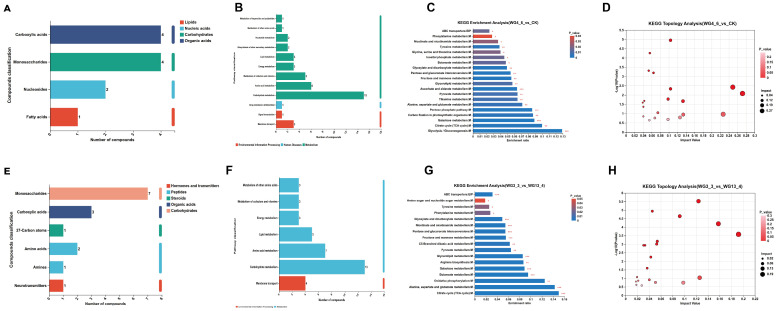
KEGG Brite classification and metabolic pathway enrichment of differentially abundant metabolites (DAMs) in Orah leaf samples. (**A**) KEGG Brite (Level 1/2) compound classification of DAMs between healthy (CK) and Huanglongbing (HLB)-infected (WG4_6) Orah leaves. (**B**) KEGG Brite (Level 1/2) metabolic pathway classification of DAMs for CK vs. WG4_6. Level 1 categories cover environmental information processing, human diseases, and metabolism; the top Level 2 categories are carbohydrate metabolism, amino acid metabolism, and coenzyme and vitamin metabolism. (**C**) KEGG enrichment analysis of DAMs for CK vs. WG4_6 (adjusted *p <* 0.05). A total of 20 pathways are significantly enriched, including galactose metabolism, ascorbate and aldarate metabolism, glycolysis/gluconeogenesis, and pentose phosphate pathway (marked with/for significance). The color gradient represents *p*-value, and bar length denotes enrichment ratio. (**D**) KEGG topology analysis of DAMs for CK vs. WG4_6, integrating *p*-value (color gradient) and pathway impact value (dot size) to assess the biological relevance of enriched pathways. (**E**) KEGG Brite (Level 1/2) compound classification of DAMs between HLB-infected Orah leaves from distinct orchards (WG3_3 vs. WG13_4). Level 1 categories consist of 5 classes, with monosaccharides and carboxylic acids as the most annotated metabolite classes. (**F**) KEGG Brite (Level 1/2) metabolic pathway classification of DAMs for WG3_3 vs. WG13_4. Level 1 categories include environmental information processing and metabolism; the top Level 2 categories are amino acid metabolism and carbohydrate metabolism. (**G**) KEGG enrichment analysis of DAMs for WG3_3 vs. WG13_4 (adjusted *p <* 0.05). (**H**) KEGG topology analysis of DAMs for WG3_3 vs. WG13_4, visualizing pathway importance via *p*-value (color gradient) and impact value (dot size). Asterisks indicate levels of statistical significance: * *p* < 0.05, ** *p*< 0.01, *** *p* < 0.001.

KEGG Brite Level 1 categories for healthy vs. diseased leaves included carbohydrates, lipids, nucleic acids, and organic acids, with monosaccharides and carboxylic acids as the most annotated metabolites (Figure 6A). KEGG metabolic pathways (Level 1) covered environmental information processing, human diseases, and metabolism, with carbohydrate metabolism, amino acid metabolism, and coenzyme and vitamin metabolism as the top Level 2 categories (Figure 6B, Table S12). After MapMan classification (*p <* 0.05), differential metabolites were assigned to 20 significantly enriched pathways, including galactose metabolism, ascorbate and aldarate metabolism, glycolysis/gluconeogenesis, and pentose phosphate pathway (Figure 6C,D; Table S13). For diseased leaves from different orchards, KEGG Brite Level 1 categories included 5 classes, with monosaccharides and carboxylic acids as the most annotated metabolites (Figure 6E). KEGG metabolic pathways (Level 1) covered environmental information processing and metabolism, with amino acid metabolism and carbohydrate metabolism as the top Level 2 categories (Figure 6F, Table S14). Differential metabolites were involved in 17 significantly enriched pathways (*p <* 0.05), including alanine, aspartate and glutamate metabolism, galactose metabolism, butanoate metabolism, citrate cycle (TCA cycle), and glycerolipid metabolism (Figure 6G,H; Table S15).

### 2.8. Integrated Analysis of Dual-Platform Data

Specific potential biomarkers were identified from each platform: 6 from LC-MS (neoglycyrrhizin 2′-apigenin glycoside, 1-methoxy-3-(4-hydroxyphenyl)-2E-propenyl 4′-glucoside, acetophenone 6′-[xylosyl-(1→6)-glucoside], quinic acid, kaempferol 3-O-glucosyl-(1→2)-rhamnoside, sucrose) and 7 from GC-MS (isothreonic acid, 4-methyl-2-penten-2,4-diol, 1,1-di-C-octyl-D-glucitol, 3-hydroxyisovaleric acid, trans-hex-2-enoic acid, inositol).

Combined KEGG pathway enrichment analysis showed that differential metabolites from both platforms were co-enriched in two core metabolic pathway categories: (1) carbohydrate metabolic pathways (starch and sucrose metabolism, map00500; galactose metabolism, map00052); (2) amino acid metabolic pathways (phenylalanine metabolism, map00360; alanine-aspartate-glutamate metabolism, map00250).

## 3. Discussion

This study innovatively integrated LC-MS and GC-MS metabolomic platforms, leveraging their complementary strengths in detecting polar and non-polar metabolites. The scientific validity of this strategy is supported by pioneering studies on integrated dual-platform data fusion frameworks, which effectively reveal primary–secondary metabolic crosstalk and improve the comprehensiveness and accuracy of metabolite identification [18]. The specificity of HLB-characteristic metabolites was further verified by mass spectrometry imaging, with spatial distribution highly consistent with pathogen-infected regions [19], providing multi-technical cross-validation for biomarker reliability.

Current metabolomic approaches for Huanglongbing (HLB) research present notable technical limitations, as the vast majority of studies rely exclusively on single liquid chromatography-mass spectrometry (LC-MS) platforms. Although LC-MS performs well in detecting semi-polar and polar secondary metabolites, it exhibits clear limitations in capturing volatile small molecules, primary metabolites, and non-polar compounds [16]. In contrast, gas chromatography-mass spectrometry (GC-MS) offers unique advantages in analyzing low-molecular-weight primary metabolites, enabling accurate quantification of key compounds such as organic acids, carbohydrates, and amino acids that directly reflect plant energy status and stress response levels.

To overcome the deficiencies of single-platform analysis, this study established an integrated LC-MS/GC-MS dual-platform metabolomic workflow and applied it to explore the metabolic mechanisms underlying HLB infection in Orah mandarin. In total, 1229 metabolites, dominated by secondary metabolites, were annotated using LC-MS, whereas 175 primary metabolites were detected via GC-MS. Notably, only one common candidate biomarker—1,1-di-C-octyl-D-glucitol—was identified across both platforms. This result directly demonstrates the strong complementarity of the two techniques and indicates that leading to an incomplete understanding of global metabolic reprogramming induced by HLB.

Compared with previously reported single LC-MS studies in sweet orange [15], our dual-platform approach provides more comprehensive metabolite coverage and enhances the sensitivity and reliability of biomarker screening. More importantly, carbohydrate metabolism and amino acid metabolism pathways were both significantly enriched in the results from both platforms. Such cross-platform consistency provides robust support for the core metabolic signatures of HLB and effectively reduces false positives caused by platform-specific technical biases.

Six flavonoid and carbohydrate-specific biomarkers identified by LC-MS are consistent with the reported defensive roles of flavonoids in HLB-tolerant citrus cultivars [20]. Seven organic acid and alcohol-specific biomarkers screened by GC-MS also match the differential expression of organic acids in HLB-infected citrus juice [21]. Together, these biomarkers cover multi-dimensional HLB metabolic characteristics, overcoming the limitations of single-platform metabolite capture and laying a foundation for constructing a multi-index combined diagnostic system.

The 13 potential biomarkers identified in this study can be classified into three functional categories associated with distinct aspects of HLB pathogenesis. First, defense response-related biomarkers, including neoglycyrrhizin 2′-apigenin glucoside, kaempferol 3-O-glucosyl-(1→2)-rhamnoside, and quinic acid, are key components of the phenylpropanoid pathway, which constitutes the core plant defense pathway against pathogen infection. Hijaz et al. reported that flavonoid accumulation was significantly higher in HLB-tolerant citrus cultivars [20], and our results further confirm that these flavonoid glycosides are specifically upregulated in HLB-infected Orah mandarin leaves, suggesting that Orah mandarin activates the phenylpropanoid defense pathway in response to CLas infection. Second, energy metabolism-related biomarkers, including sucrose, inositol, and ribitol, are important intermediates in carbohydrate metabolism. The prominent accumulation of sucrose in infected leaves is consistent with the classic “phloem blockage” hypothesis of HLB, in which CLas infection impairs phloem transport, leading to carbohydrate accumulation and subsequent leaf chlorosis [22]. Notably, inositol, identified as a GC-MS biomarker in this study, has not been reported in previous sweet orange studies, indicating its potential as a cultivar-specific biomarker for Orah mandarin. Third, stress response-related biomarkers, including 3-hydroxyisovaleric acid, trans-hex-2-enoic acid, and 1,1-di-C-octyl-D-glucitol, are involved in plant oxidative stress and membrane lipid metabolism. Among these, 1,1-di-C-octyl-D-glucitol was the only cross-platform biomarker detected in both LC-MS and GC-MS, and its content was significantly elevated in all infected samples regardless of orchard origin. This high stability renders it the most promising candidate biomarker for universal early diagnosis of HLB.

Cross-study comparisons revealed considerable differences in HLB-related metabolic biomarkers among different citrus cultivars. For instance, key biomarkers identified in sweet orange, such as limonin and naringin [15], were not detected as differential metabolites in Orah mandarin. Similarly, inositol, a biomarker specific to Orah mandarin identified in this study, has not been reported in sweet orange. Such cultivar specificity accounts for the low diagnostic accuracy of existing HLB detection methods when directly applied from sweet orange to Orah mandarin, and underscores the importance of developing cultivar-specific diagnostic biomarkers.

Dual-platform differential metabolites were co-enriched in carbohydrate metabolic pathways (starch-sucrose metabolism, galactose metabolism) and amino acid metabolic pathways (phenylalanine metabolism, alanine-aspartate-glutamate metabolism), which is consistent with HLB metabolomic reviews identifying these as the most affected core pathways [22], further validating the conserved pattern of HLB-induced citrus core metabolic disorders.

Based on our dual-platform metabolomic results, we propose a three-stage metabolic pathogenesis model of HLB in Orah mandarin. In the early stage, CLas colonizes phloem sieve elements and induces callose deposition, leading to phloem blockage [1]. This disturbance impairs the long-distance translocation of photosynthetic products, resulting in substantial sucrose and starch accumulation in leaves, as supported by the significant enrichment of starch and sucrose metabolism (map00500). Excessive carbohydrate accumulation further inhibits photosynthesis, causing insufficient energy supply for plant growth and the appearance of typical mottled chlorosis symptoms.

In the middle stage, plants undergo amino acid metabolic reprogramming to alleviate energy deficiency and resist pathogen infection. Alanine, aspartate, and glutamate metabolism (map00250) is significantly disrupted, which is consistent with observations reported by Suh et al. [23] in HLB-tolerant citrus cultivars. As a central hub in amino acid metabolism, glutamate serves not only as a critical energy substrate under stress but also as a precursor for the biosynthesis of defense-related secondary metabolites. The upregulation of phenylalanine metabolism (map00360) further indicates that plants redirect carbon flux from primary metabolism toward secondary defense metabolism to counteract CLas infection.

In the late stage, long-term imbalance between energy supply and defense responses triggers reactive oxygen species (ROS) accumulation and oxidative damage. Pronounced changes in organic acids (e.g., lactic acid and hexanoic acid) and alcohols (e.g., ribitol and inositol) detected by GC-MS serve as direct indicators of oxidative stress and membrane lipid peroxidation [24]. The cumulative damage ultimately leads to leaf abscission, fruit drop, and tree decline.

This model clarifies the causal relationship between carbohydrate and amino acid metabolic disorders and illustrates the dynamic progression of HLB pathogenesis from a metabolic perspective. Notably, the significant enrichment of galactose metabolism across both platforms suggests that cell wall metabolism may also serve an important role in HLB infection, which has been largely overlooked in previous studies and warrants further research.

Notably, alanine-aspartate-glutamate metabolism disruption has been independently confirmed by targeted metabolomic comparisons of HLB-tolerant vs. susceptible cultivars [23], and global amino acid metabolic abnormalities are a typical HLB infection feature [24]. Building on this, our study further reveals the synergistic interference of HLB on plant energy and defense metabolism, deepening the understanding of HLB pathogenesis.

This cross-platform validation enhances the credibility and diagnostic potential of candidate biomarkers via literature-validated core pathway targets and provides clear directions for deciphering citrus HLB-resistant metabolic regulatory networks. It achieves an organic combination of technical innovation and biological mechanism exploration, consistent with the application paradigm of dual-platform metabolomics in plant disease research. Despite these noteworthy findings, several critical limitations of this study should be acknowledged. First, the sample size used for metabolomic analysis is relatively limited, including only 9 HLB-positive Orah mandarin samples collected from Wuming District, Nanning. Although infected samples from Liuzhou were also analyzed, the insufficient number of Orah mandarin specimens may restrict the generalizability of the results. In addition, all samples were collected at a single time point, which fails to reflect dynamic metabolic changes throughout the full infection cycle of HLB from asymptomatic to severe stages.

Second, metabolomic profiling based on whole-leaf samples may dilute HLB-specific metabolic signals, because CLas is unevenly distributed and predominantly colonizes phloem tissues. Future studies using laser microdissection for isolating pure phloem tissues or mass spectrometry imaging to visualize spatial metabolite distribution will help identify more specific and sensitive biomarkers [19].

Third, metabolite levels are susceptible to environmental factors, including temperature, rainfall, soil fertility, and orchard management practices. Although orchards with similar management regimes were selected, environmental interference could not be fully eliminated, as reflected by the distinct metabolic profiles among infected samples from different orchards. Accordingly, the diagnostic thresholds of candidate biomarkers may need to be adjusted according to regional and cultivation conditions.

Finally, all candidate biomarkers identified in this study were screened based on statistical correlations, and their direct functional roles in HLB pathogenesis remain to be experimentally verified. Further studies involving gene editing or exogenous metabolite treatment are needed to clarify whether these metabolites contribute to HLB symptom development or merely represent secondary responses.

Based on the 13 potential biomarkers identified in this study, subsequent research should focus on their systematic validation and translational application. For validation, efforts should be made to expand the sample size; samples should cover the complete pathological gradient from healthy, early asymptomatic, and mild to severe huanglongbing (HLB) infection, with related citrus varieties included as controls. Targeted metabolomics will be employed for accurate quantitative analysis of candidate biomarkers to determine their expression thresholds at different disease stages, with a focus on verifying their efficacy in identifying early asymptomatic plants. This will help eliminate interference from environmental heterogeneity and improve the specificity and reliability of candidate biomarkers. Additionally, indoor artificial inoculation experiments mediated by viruliferous *Diaphorina citri* can be conducted to track the temporal dynamic changes in candidate biomarkers following *Candidatus Liberibacter asiaticus* (CLas) infection, clarifying their correlation with HLB progression and providing robust data support for subsequent application research.

In terms of translational application, priority should be given to the development of low-cost and easy-to-operate detection technologies. Based on highly specific candidate biomarkers, enzyme-linked immunosorbent assay (ELISA) kits or colloidal gold immunochromatographic test strips can be developed. We aim to develop a colloidal gold immunochromatographic test strip for rapid, on-site detection. Such a test strip would enable simple, instrument-free screening under field conditions, making it suitable for large-scale preliminary detection of HLB by grassroots agricultural technicians without the need for sophisticated laboratory facilities. Optimization of detection sensitivity will ensure operability by grassroots technicians after simple training, enabling rapid preliminary screening of field samples. Furthermore, validated biomarkers can serve as reference indicators for screening HLB-resistant *Citrus reticulata* ‘Orah’ germplasm. For disease-resistant breeding, we will establish a high-throughput biomarker-based screening system to identify citrus germplasm with stable resistance to HLB. The resistant germplasm identified in this way will be used as parental lines to breed new HLB-resistant Orah mandarin varieties with desirable horticultural and fruit quality traits. For major cultivars and local germplasm resources in various citrus-producing regions, strains with strong disease resistance can be selected by detecting biomarker expression levels, providing parental materials for disease-resistant breeding. Meanwhile, combined with field plot experiments, the dynamic changes in biomarkers can be used to evaluate the effectiveness of common control measures (e.g., application of virus-free seedlings, green control of *Diaphorina citri*), offering data references for formulating region-specific HLB control strategies and facilitating HLB prevention and control in the global citrus industry.

As Guangxi represents the largest citrus-producing region in China, where Orah mandarin accounts for more than 30% of the total citrus cultivation area and HLB causes annual economic losses exceeding 1 billion Chinese Yuan [17], this study offers a robust scientific basis for establishing practical HLB prevention and control strategies. The biomarkers and metabolic pathways identified herein will not only improve the accuracy of early HLB diagnosis but also support the development of innovative control strategies such as metabolic regulation to enhance citrus resistance to HLB, thereby contributing to the sustainable development of the global citrus industry.

## 4. Materials and Methods

### 4.1. Plant Materials and Treatment

Leaf samples were collected from eight citrus cultivars, namely Orah mandarin, seedless Orah mandarin, Shatangju mandarin, tangelo, Satsuma mandarin, sweet orange, Golden Autumn mandarin, and Nanfeng tangerine; fully expanded healthy leaves with uniform tree vigor and leaf age were selected, while diseased leaves exhibited typical HLB symptoms (mottled chlorosis and red-nosed fruit). A total of 700 suspected HLB-infected leaf samples were collected and photographed from commercial orchards in Nanning, Liuzhou, and the experimental orchard of Guangxi University in the Guangxi Zhuang Autonomous Region, among which the confirmed positive samples were distributed as follows: 11 from Satsuma mandarin (sample code WZ), 8 from sweet orange (sample code TC), 11 from Shatangju mandarin (sample code ST), 12 from Nanfeng tangerine (sample code NF), 9 from Orah mandarin (sample code WG), and 11 from tangelo (sample code JY). Field symptom observation was only used for preliminary screening, and all candidate samples were finally confirmed for *Candidatus Liberibacter asiaticus* (CLas) infection via targeted qPCR to ensure sample accuracy. After collection, the samples were wrapped in slightly moist sterile filter paper for short-distance transportation (approximately 2 h), a measure that prevented excessive leaf dehydration without directly wetting the leaves, thereby avoiding metabolic disturbance. All samples were immediately snap-frozen in liquid nitrogen upon collection, temporarily stored at −20 °C during short-term transportation, and uniformly stored long-term at −80 °C for metabolomic analysis to prevent the degradation of temperature-sensitive metabolites, ensure consistent preservation conditions for all samples, and minimize inter-group variation. To exclude confounding effects of environmental and agronomic conditions, we clearly documented the geographical origins (Wuming, Nanning, and Liuzhou), climate types, and field management practices of the sampling orchards; in subsequent metabolomic analyses, only Orah mandarin samples were used to maximize the reduction in metabolic background differences caused by variety, region, and management, though environmental heterogeneity among different orchards may still have a certain impact on metabolic profiles, which has been considered in the interpretation of results.

### 4.2. DNA Extraction

Total genomic DNA was extracted from citrus leaf midrib tissues using the CTAB method [25]. Tissues were ground into fine powder in liquid nitrogen, followed by addition of extraction buffer and thorough vortexing. The mixture was centrifuged at 12,000 rpm for 5 min, the supernatant was discarded, and the step was repeated once. Subsequently, extraction buffer, lysis buffer, and RNase were added to the pellet, vortexed thoroughly, and incubated in a 65 °C water bath for 30–45 min (with occasional inversion). The mixture was sequentially extracted with chloroform:isoamyl alcohol (24:1, *v*/*v*) and phenol:chloroform:isoamyl alcohol (25:24:1, *v*/*v*), with each extraction followed by centrifugation at 12,000 rpm for 5 min. The supernatant was collected, mixed with an equal volume of isopropanol, and centrifuged at 12,000 rpm for 15 min to obtain the DNA pellet. The pellet was washed with 70% ethanol, centrifuged, air-dried at room temperature, dissolved in TE buffer, and stored at −20 °C until use.

Extracted DNA was analyzed by 1% agarose gel electrophoresis, showing clear bands without smearing. Nanodrop spectrophotometer analysis revealed an A_260_/A_280_ ratio of 1.8–2.0, confirming that DNA integrity and purity met the requirements for quantitative real-time PCR (qPCR).

### 4.3. Quantitative Real-Time PCR (qPCR) Assay

qPCR assays were performed in 96-well plates sealed with optical films (to prevent contamination and evaporation) using an Eppendorf real-time PCR system. For DNA extraction, 0.1 g leaf midrib and 0.2 g pericarp tissues were sampled following standard protocols to avoid cross-contamination. The 20 μL total reaction volume contained 10.0 μL TaKaRa Premix ExTaq (2×), 0.5 μL each of 10 μmol/L primers HLBas and HLBr, 0.5 μL 10 μmol/L probe HLBp, 7.5 μL double-distilled water (ddH_2_O), and 1.0 μL template DNA. For reliability and reproducibility, each sample had 3 technical replicates, with a no-template control (NTC, ddH_2_O instead of template) to exclude cross-contamination and reagent background. The standard template (initial 24 ng/μL) was 10-fold serially diluted to 5 concentrations (10^1^–10^5^ folds) for standard curve construction. The qPCR program was as follows: pre-denaturation at 95 °C for 30 s, followed by 40 cycles of 95 °C denaturation for 5 s and 60 °C annealing/extension for 30 s [26].

### 4.4. Untargeted Metabolite Extraction and LC-MS Quality Control

Forty-two citrus leaf samples were divided into 7 groups (CK, WG13_4, WG3_3, WG13_6, WG4_6, WG5_9, WG5_1), with 6 biological replicates per group. For sample preparation, 50 mg leaf samples were weighed and mixed with 400 μL extraction solution (methanol:water = 4:1, *v*/*v*). Samples were homogenized at −20 °C and 50 Hz for 6 min using a high-throughput tissue homogenizer, vortexed for 30 s, followed by low-temperature ultrasonic extraction at 5 °C and 40 KHz for 30 min. After standing at −20 °C for 30 min to precipitate proteins, samples were centrifuged at 4 °C and 13,000 *g* for 15 min. The supernatant was transferred to LC-MS vials for analysis [27].

For system conditioning and quality control (QC), a pooled QC sample was prepared by mixing equal volumes of all samples, processed and detected identically to analytical samples to represent the entire sample set. One QC sample was inserted every 6 analytical samples to monitor instrument stability and analytical reliability.

### 4.5. UHPLC-MS/MS Analysis

Analyses were conducted on a Thermo Scientific Vanquish Horizon ultra-high performance liquid chromatography (UHPLC) system coupled with a Q-Exactive mass spectrometer [28]. An ACQUITY BEH C_18_ column (100 mm × 2.1 mm i.d., 1.7 μm, Waters, Milford, MA, USA) was used. The mobile phase consisted of solvent A (water with 0.1% formic acid) and solvent B (acetonitrile/isopropanol = 1:1, *v*/*v*, 0.1% formic acid). The injection volume was 10 μL, the flow rate was 0.4 mL/min, and the column temperature was maintained at 40 °C.

Identical solvent gradient programs were applied for positive and negative ion modes: 95% A/5% B at 0 min; 80% A/20% B at 3 min; 5% A/95% B at 9 min; held at 5% A/95% B (9–13 min); returned to 95% A/5% B at 13.1 min; held until 16 min for system equilibration. All samples were stored at 4 °C prior to analysis.

MS data were acquired via an electrospray ionization (ESI) source, operating in both positive and negative ion modes. Operating conditions: mass range *m*/*z* 70–1050; sheath gas flow 40 psi, auxiliary gas flow 10 psi; ion source temperature 400 °C; ion spray floating voltage (ISVF) −2800 V (negative mode) and 3500 V (positive mode); MS/MS collision energy normalized with rolling voltages of 20–40–60 V. Total ion chromatograms (TICs) obtained under these conditions exhibited good peak shapes and uniform distribution.

### 4.6. LC/MS Data Processing and Annotation

Raw LC-MS data were preprocessed with Progenesis QI software (version 2.4, Waters Corporation, Milford, MA, USA), involving baseline filtering, peak detection, integration, retention time correction, and peak alignment. A three-dimensional CSV data matrix containing sample information, metabolite names, *m*/*z* values, retention time, and spectral response intensity was exported afterwards. During data processing, internal standard peaks were introduced and eliminated to calibrate systematic errors, and L-2-chlorophenylalanine was applied as the internal standard to monitor sample pretreatment stability, chromatographic retention time repeatability, and instrument analytical performance, thus guaranteeing the credibility of qualitative and quantitative results. Meanwhile, internal standard signals, solvent peaks, column bleed, and other known false positive peaks were also discarded [29].

Metabolite annotation and identification were conducted based on accurate mass matching with mass deviation less than 10 ppm, retention time regularity, isotopic distribution patterns, and MS/MS fragment alignment. Relevant databases, including KEGG, HMDB, METLIN, and an in-house standard substance database, were adopted for spectrum matching. The confidence level of identified metabolites was graded in line with the guidelines of the Metabolomics Standards Initiative (MSI) to enhance the scientific rigor and repeatability of untargeted metabolomic identification. All processed datasets were further analyzed on the Majorbio Cloud Platform.

Data preprocessing: Metabolic features with non-zero values in ≥80% of samples in at least one group were retained. Missing values below the limit of quantitation were imputed with the minimum value. Each metabolic feature was normalized by total peak normalization (to correct sample preparation errors caused by large metabolite polarity differences and significant peak intensity fluctuations in LC-MS detection). Variables with relative standard deviation (RSD) ≥ 30% in QC samples were removed to obtain the final data matrix for subsequent analysis [30].

### 4.7. Untargeted Metabolite Extraction and GC-MS Quality Control

A total of 42 citrus leaf samples were used in this study, with the same grouping design as that for LC-MS analysis and six biological replicates per group. For sample preparation, 50 mg of leaf tissue was weighed into 2 mL centrifuge tubes, followed by the addition of 500 μL extraction solution (methanol:water = 4:1, *v*/*v*) and one steel bead; samples were then homogenized (ground) at low temperature for 3 min at 50 Hz using a high-throughput tissue homogenizer, after which 200 μL chloroform was added and homogenization was continued for another 3 min under the same conditions. After vortex mixing, ultrasonic extraction was performed in an ice-water bath three times (10 min each), and samples were then incubated at −20 °C for 30 min to precipitate proteins, followed by centrifugation at 4 °C and 12,000 rcf for 20 min; the resulting supernatant was transferred to glass derivatization vials and vacuum-dried. For derivatization, 80 μL methoxyamine hydrochloride pyridine solution (15 mg/mL) was added, vortexed for 2 min, and incubated at 37 °C for 90 min for oximation; subsequently, 80 μL BSTFA derivatizing reagent (containing 1% TMCS) and 20 μL n-hexane were added, vortexed for 2 min, reacted at 70 °C for 60 min, and allowed to stand at room temperature for 30 min before GC-MS analysis [31]. For system conditioning and quality control, L-2-chlorophenylalanine was added as an internal standard prior to metabolite extraction to monitor extraction efficiency, derivatization stability, and instrumental repeatability throughout the entire experiment; in addition, equal volumes of all samples were mixed to prepare pooled QC samples, and one QC sample was injected every six test samples during the analytical run to evaluate the stability of the GC-MS analysis system and the reliability of the data.

### 4.8. GC-MS Analysis

Analyses were conducted on an Agilent 8890B-5977B gas chromatography-mass spectrometry (GC-MS) system (Agilent Technologies, Santa Clara, CA, USA). An HP-5MS UI capillary column (30 m × 0.25 mm i.d., 0.25 μm film thickness, Agilent J&W Scientific, Santa Clara, CA, USA) was used. Derivatized samples were injected in split mode (split ratio 10:1) with an injection volume of 1 μL. High-purity helium served as the carrier gas at 1 mL/min, septum purge flow 3 mL/min, and solvent delay 5 min. Column temperature program: initial 60 °C for 0.5 min, ramped to 310 °C at 8 °C/min, and held for 6 min. All samples were stored at room temperature prior to analysis.

MS data were acquired via an electron impact ionization (EI) source in full scan mode. Operating conditions: electron energy 70 eV; ion source temperature 230 °C; quadrupole temperature 150 °C; mass range *m*/*z* 50–500; scan frequency 3.2 scan/s. TICs obtained under these conditions exhibited satisfactory peak shapes and uniform distribution.

### 4.9. GC-MS Data Processing and Annotation

Raw GC-MS data were processed using ChromaTOF software (v4.34, LECO, St Joseph, MI, USA), and a 3D CSV data matrix (sample information, metabolite names, retention time, quantitative ions, mass spectral response intensity) was exported. Internal standard peaks and known false-positive peaks (noise, column bleed, derivatization reagent peaks) were removed, followed by deduplication and peak merging.

Metabolite identification was conducted via KEGG and HMDB databases; metabolites with a matching score ≥80% were reliably identified. All data were uploaded to the Majorbio Cloud Platform (https://cloud.majorbio.com, accessed on 18 September 2019) for further analysis.

Data preprocessing: Missing values were imputed with the minimum value of each column in the raw matrix. Each metabolic feature was normalized by total sum normalization (to retain relative content relationships of GC-MS-detected volatile small-molecule metabolites). Variables with RSD ≥ 30% in QC samples were removed, followed by log_10_ transformation to obtain the final data matrix for subsequent analysis.

### 4.10. Differential Metabolite Analysis

Multivariate statistical analyses, including principal component analysis (PCA) and orthogonal partial least squares discriminant analysis (OPLS-DA), were performed using the ropls package (Version 1.6.2) in R language. PCA was used to observe overall sample distribution and intergroup dispersion, with R^2^X > 0.5 (model explanation rate) as the validity criterion. OPLS-DA filtered out “noise” variables in the observation matrix X unrelated to the prediction matrix Y, improving intergroup discrimination and model interpretability. Metabolite annotation was referenced to the HMDB and KEGG databases, and pathway enrichment analysis was conducted using the scipy.stats package in Python(version.1.0.0).

Differential metabolites were screened via *t*-test combined with OPLS-DA, requiring variable importance in projection (VIP) > 1 and *p <* 0.05 to reduce false positives from multiple testing.

Correlation analysis assessed intragroup reproducibility and intergroup variability. The KEGG database was used for metabolite and pathway classification. Cluster analysis and heatmaps visualized differential metabolite expression patterns across groups. Pathway enrichment analysis and KEGG-based metabolic mapping linked metabolite differences to biochemical pathways, identifying key involved metabolic processes.

Dual-platform data integration identified core differential metabolites meeting screening criteria in both GC-MS and LC-MS, further enhancing result reliability.

## Figures and Tables

**Table 1:** Citrus cultivars and corresponding sampling locations.

Cultivar	Sampling Location
Satsuma mandarin	Liubei District, Liuzhou
Sweet orange	Liubei District, Liuzhou
Shatangju mandarin	Liubei District, Liuzhou
Nanfeng tangerine	Liubei District, Liuzhou
Orah mandarin	Wuming District, Nanning

## Data Availability

All data are available in the manuscript or the Supplemental Materials. All other data and materials generated in this study are available from the corresponding authors upon reasonable request.

## Supplementary Materials

The following supporting information can be downloaded at: https://www.mdpi.com/article/10.3390/molecules31111873/s1, Table S1: Summary of fluorescent quantitative PCR results of all Citrus HLB; Table S2: Qualitative analysis of the different metabolites (cations) between the diseased leaves and the healthy leaves; Table S3: Qualitative analysis of the different metabolites (anions) between the diseased and healthy leaves; Table S4: Qualitative analysis of different metabolites (cations) in different orchard; Table S5: Qualitative analysis of different metabolites (anions) in different orchard; Table S6: Statistical table of KEGG pathway information in diseased and healthy leaves of Citrus-LC-MS; Table S7: Statistical table of KEGG pathway information in diseased leaves of different orchards-LC-MS; Table S8: Statistical table of KEGG Enrichment information in diseased and healthy leaves of Citrus_LC-MS; Table S9: Statistical table of KEGG Enrichment information in diseased leaves of different orchards_LC-MS; Table S10: Qualitative analysis of different metabolites in healthy and diseased leaves of Orah mandarin by GC-MS; Table S11: Qualitative analysis of different metabolites in the diseased leaves of different orchards by GC-MS; Table S12: Statistical table of KEGG pathway information in diseased and healthy leaves of Citrus-GC-MS; Table S13: Statistical table of KEGG pathway information in diseased leaves of different orchards-GC-MS; Table S14: Statistical table of KEGG Enrichment information in diseased and healthy leaves of Citrus_GC-MS; Table S15 Statistical table of KEGG Enrichment information in diseased leaves of different orchards_GC-MS.

## Abbreviations

The following abbreviations are used in this manuscript:

HLB	Huanglongbing
CLas	Candidatus Liberibacter asiaticus
GC-MS	Gas chromatography-mass spectrometry
LC-MS	Liquid chromatography-mass spectrometry
PCA	Principal component analysis
OPLS-DA	Orthogonal partial least squares-discriminant analysis
KEGG	Kyoto Encyclopedia of Genes and Genomes
DAMs	Differentially accumulated metabolites/Differentially abundant metabolites
VIP	Variable importance in projection
PCR	Polymerase Chain Reaction
LAMP	Loop-mediated isothermal amplification
UPLC-QTOF-MSE	Ultra-performance liquid chromatography-quadrupole time-of-flight mass spectrometry in MSE mode
qPCR	Quantitative real-time PCR
TIC	Total ion current
ESI	Electrospray ionization
ELISA	Enzyme-linked immunosorbent assay
CTAB	Cetyltrimethylammonium bromide
NTC	No-template control
ddH2O	Double-distilled water
UHPLC	Ultra-high performance liquid chromatography
QC	Quality control
ISVF	Ion spray floating voltage
RSD	Relative standard deviation
EI	Electron impact ionization
HMDB	Human Metabolome Database
